# Supplementary material for: RNA sequencing provides insights into the evolution of lettuce and the regulation of flavonoid biosynthesis
Source: Nat Commun. 2017 Dec 22;8:2264. doi: 10.1038/s41467-017-02445-9 (PMC5741661; doi:10.1038/s41467-017-02445-9)
Supplement: Supplementary file 1 — Supplementary Information [file 41467_2017_2445_MOESM1_ESM.pdf]

Supplementary Figures:

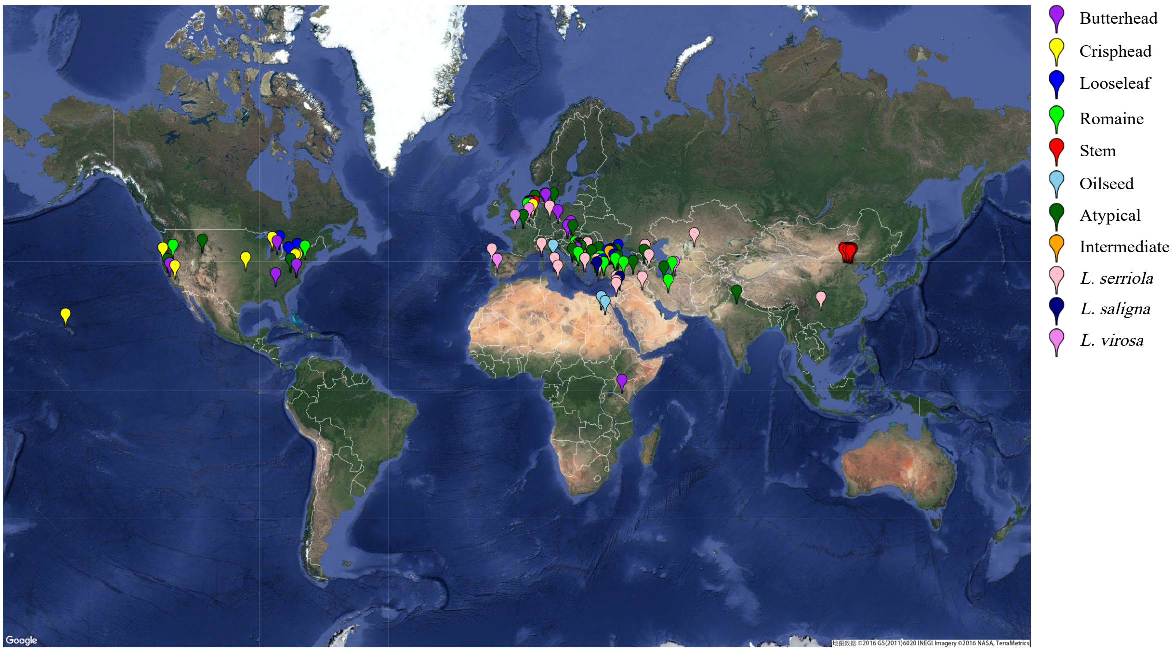

Supplementary Figure 1. Geographic distributions of the *Lactuca* accessions sampled in this study

The locations of the *Lactuca* accessions are illustrated on the Google map based on the longitudes and latitudes from either the GRIN or the CGN databases. This map was generated using the R package 'plotGoogleMaps'[1].

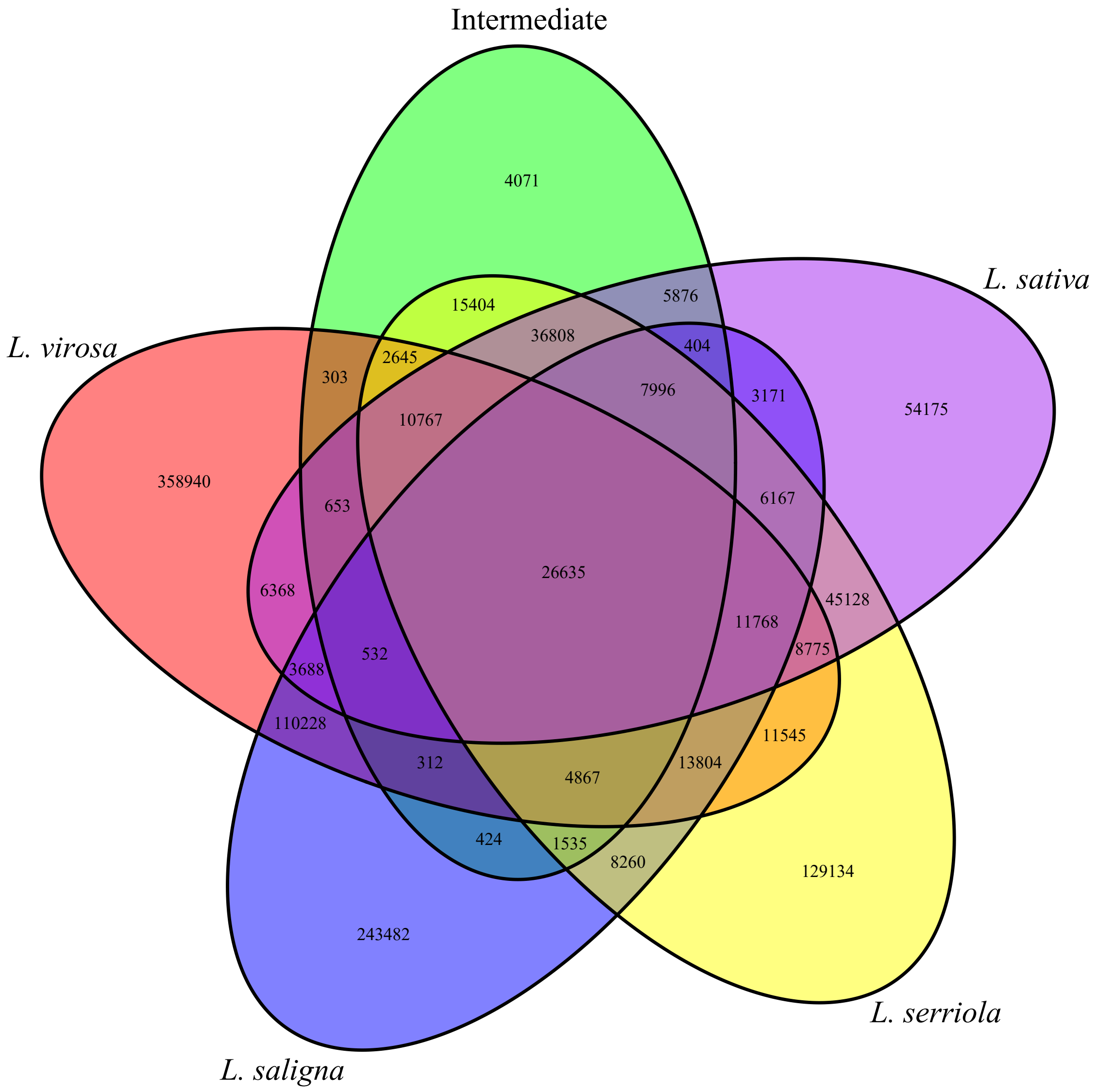

Supplementary Figure 2. Venn diagram of identified SNPs in five groups

The number of the unique SNPs in *L. virosa*, *L. saligna*, *L. serriola*, intermediate and *L. sativa* groups were 358,940, 243,482, 129,134, 4,071 and 54,175, respectively. The number of SNPs that occurred in *L. virosa* and *L. saligna* was 712,650 (62.85% of the total SNPs), indicating they are more distantly related with *L. serriola* and *L. sativa*.

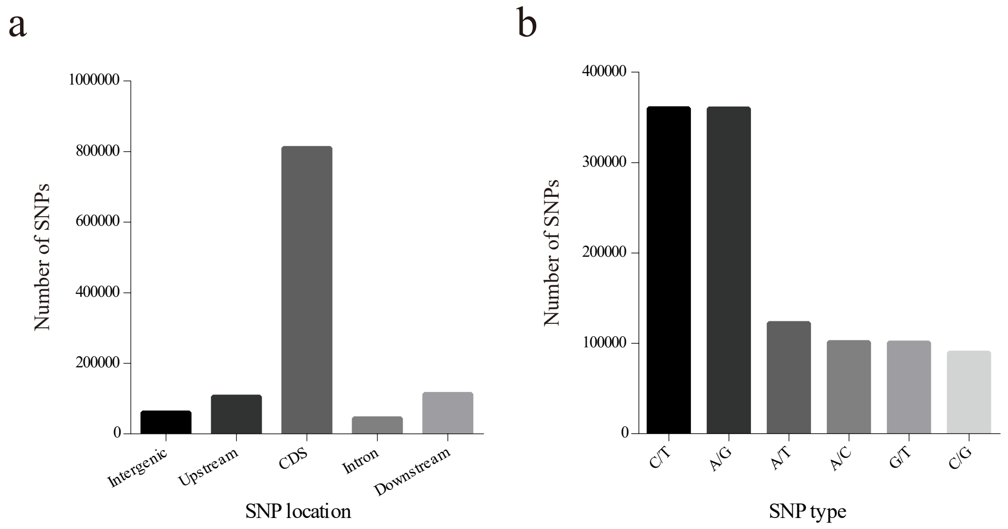

Supplementary Figure 3. Characteristics of identified SNPs

(a) Number of SNPs located in each region. Upstream refers to a region that is within 3-kb upstream of the start codon. Downstream refers to a region that is within 3-kb downstream of the stop codon. (b) Number of each SNP type.

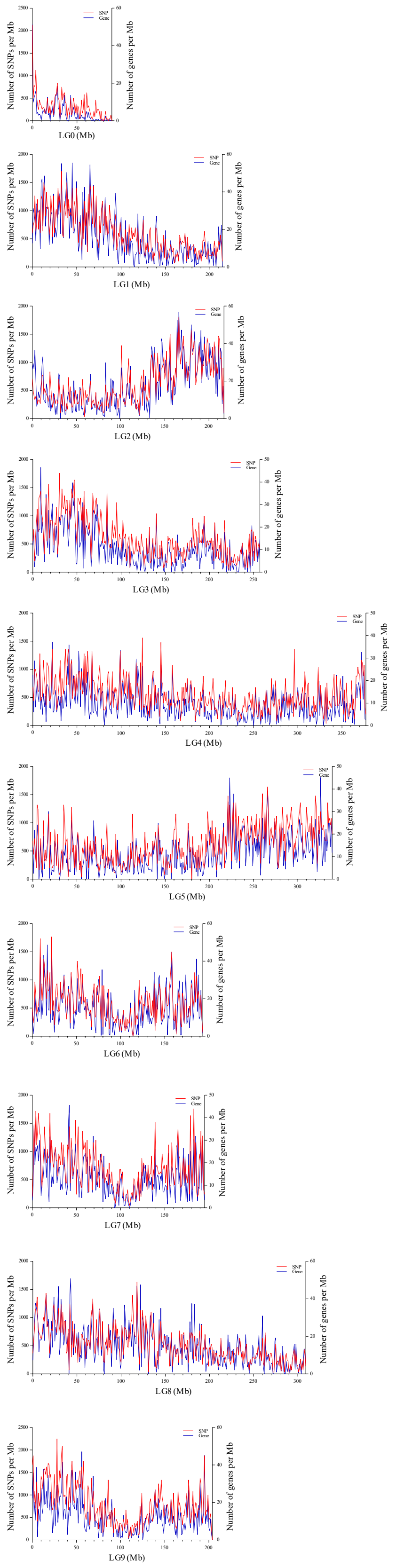

Supplementary Figure 4. Distribution of Genes and SNPs in the lettuce genome

The distribution of SNPs together with gene density were visualized across the lettuce chromosomes. A sliding window method with window size of 1 Mb was used, and we found that SNPs were not evenly distributed throughout the genome. As it can be predicted that SNP density was consistent with the density of genes, in some of the regions SNP and gene density was substantially low. These regions may be the centromere regions or gap regions that cannot anchor the scaffold on the chromosome.

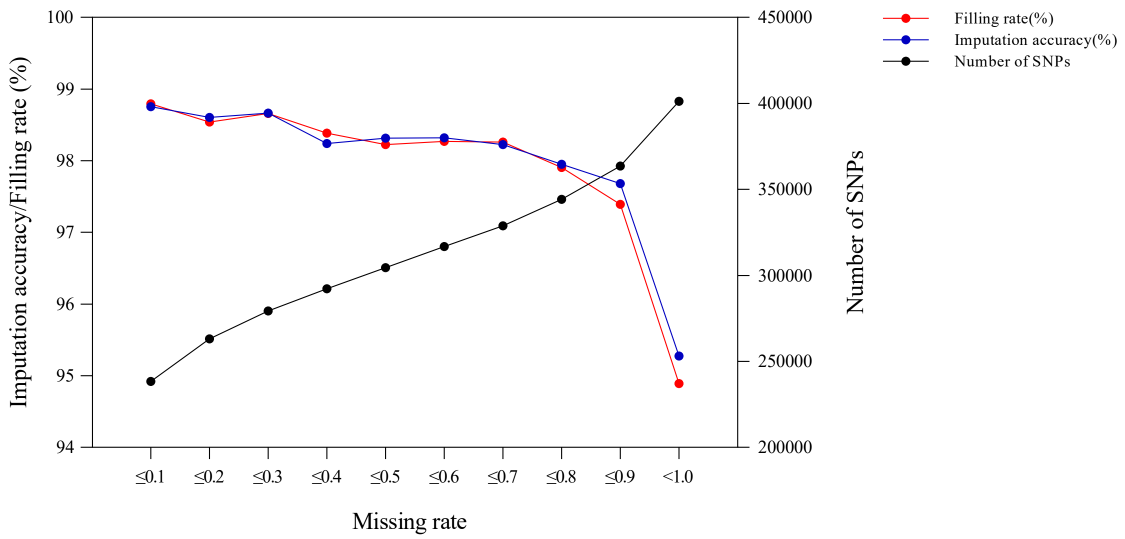

Supplementary Figure 5. Imputation accuracy, filling rate, and number of SNPs with different missing rates

The imputation accuracy was measured as the ratio of the correctly inferred genotypes to all of the masked genotypes. The filling rate was calculated as the proportion of inferred genotypes in all of the masked genotypes. The number of SNPs was counted under the certain missing genotype rate. For the missing rate that ranged from 10% to 90%, the best imputation accuracy and filling rate were chosen separately after testing 320 combinations of parameters. As indicated by the results, the optimal imputation accuracy (97.95%) and filling rate (97.90%) were achieved when the cutoff of the missing rate was set to 0.8. These parameters were then used for the final data imputation.

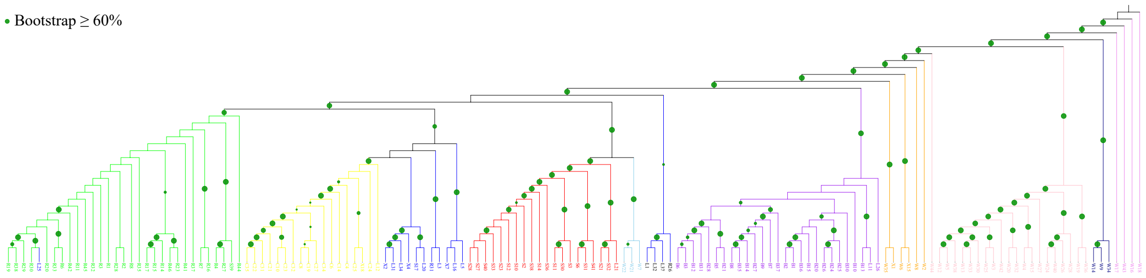

Supplementary Figure 6. Phylogenetic tree of 148 *Lactuca* accessions excluding the atypical type and RIL individuals

A maximum-likelihood phylogenetic tree was constructed using 506,821 SNP sites from 148 *Lactuca* accessions (excluding the atypical accessions and the RIL individuals). Colors correspond to the following groups: violet, *L. virosa*; navy blue, *L. saligna*; pink, *L. serriola*; orange, intermediate group; purple, butterhead; sky blue, oilseed; red, stem; green, romaine; blue, looseleaf; yellow, crisphead.

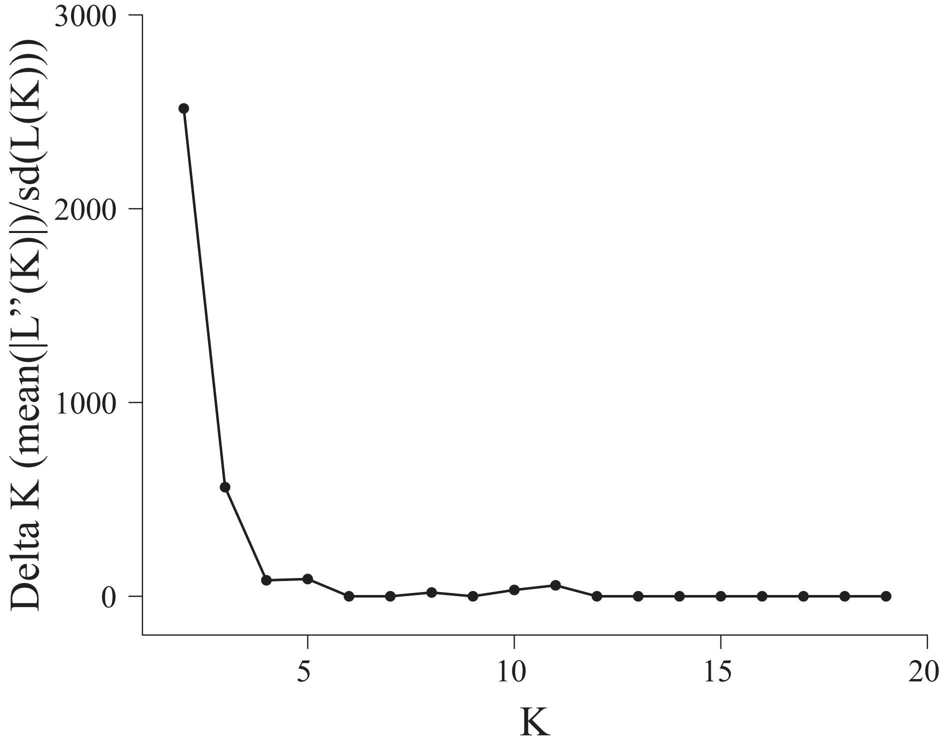

Supplementary Figure 7. ***ΔK*** **analysis for the different number of clusters for the** ***Lactuca*** **accessions**

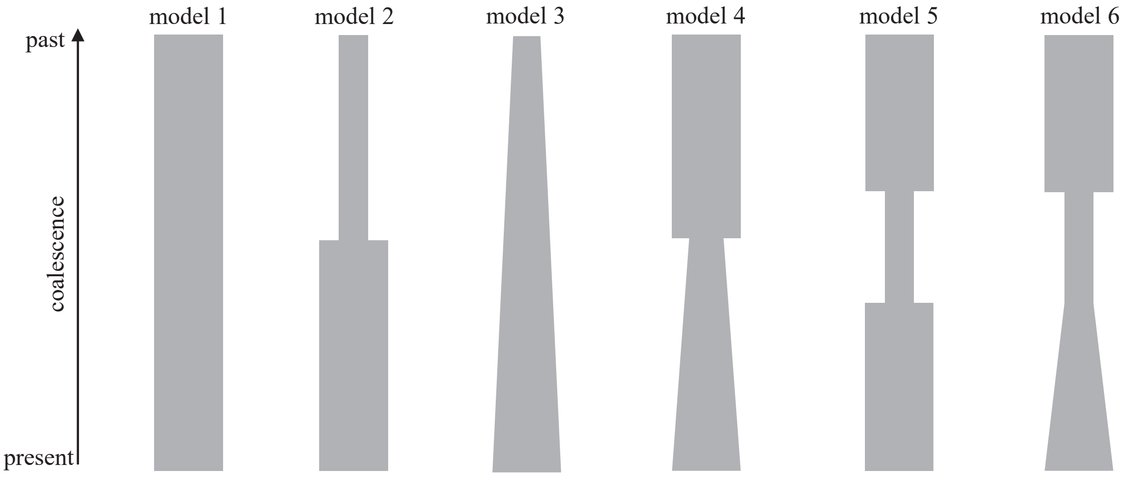

Supplementary Figure 8. Schematic diagram of six different demographic models for one-population

The six models were designed as follows: model 1, constant population size; model 2, instantaneous size change some time ago; model 3, recent exponential population growth; model 4, instantaneous size change followed by exponential growth; model 5, a three-epoch model (bottleneck of some duration followed by recovery); model 6, a three-epoch model (bottleneck of some duration followed by exponential growth). These models only indicate relative differences.

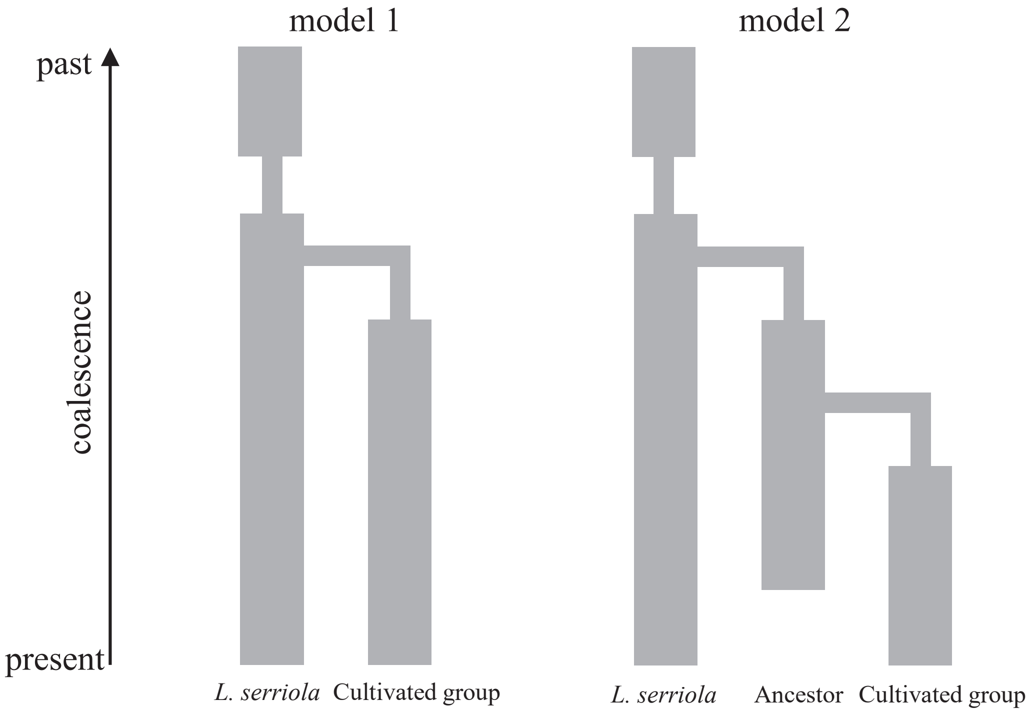

Supplementary Figure 9. Schematic diagram of two different demographic models for two-population

The two models were designed as follows: model 1, the cultivated group was originated directly from *L. serriola*; model 2, the cultivated group was originated from an ancestral cultivated group (un-sampled population in the model), and the ancestral cultivated group was originated directly from *L. serriola* (backwards in time). All of the populations were assumed to undergo demographic bottlenecks, based on the comparisons of different demographic models for one-population. These models only indicate relative differences.

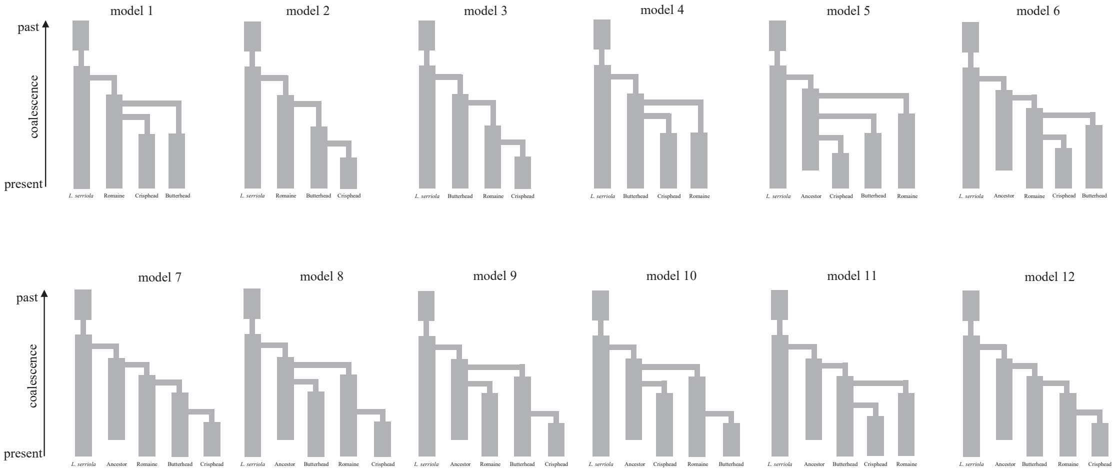

Supplementary Figure 10. Schematic diagram of 12 different demographic models for four-population

L. serriola and three leafy horticultural types (butterhead, crisphead and romaine) were analyzed with four-population models. Stem lettuce was excluded from the models because our results from PCA and STRUCTURE analyses indicated that it is the most genetically distinct group within cultivated lettuce. The ancestral cultivated group is the un-sampled population in the model. These models only indicate relative differences.

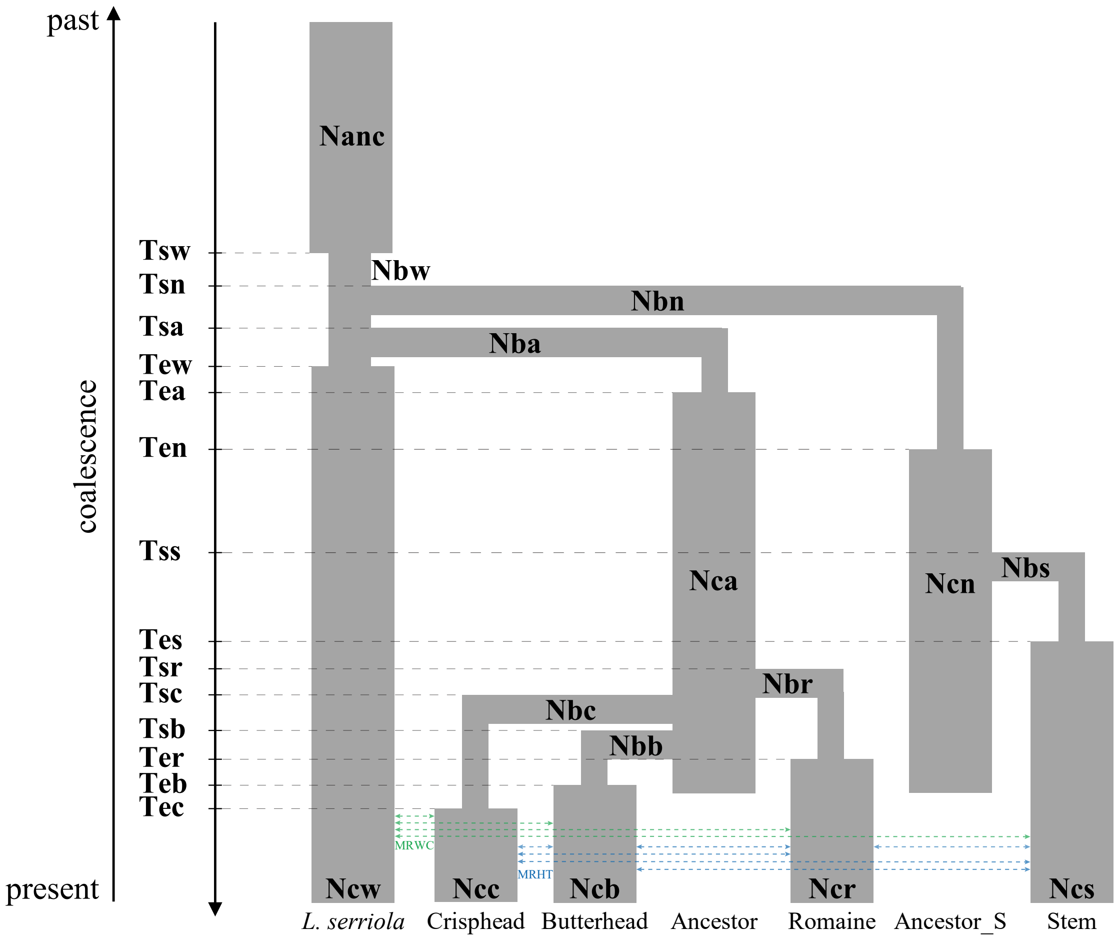

Supplementary Figure 11. Schematic diagram of double-founder demographic model for five-population

This model (double-founder model) assumes that there were two domestication events and that both stem lettuce and leafy lettuce originated independently from different ancestral cultivated populations. Gene flow among different populations was allowed. Two symmetrical migration parameters were used, one between different horticultural types (MRHT), and the other between *L. serriola* and each horticultural type (MRWC). Model only indicate relative difference.

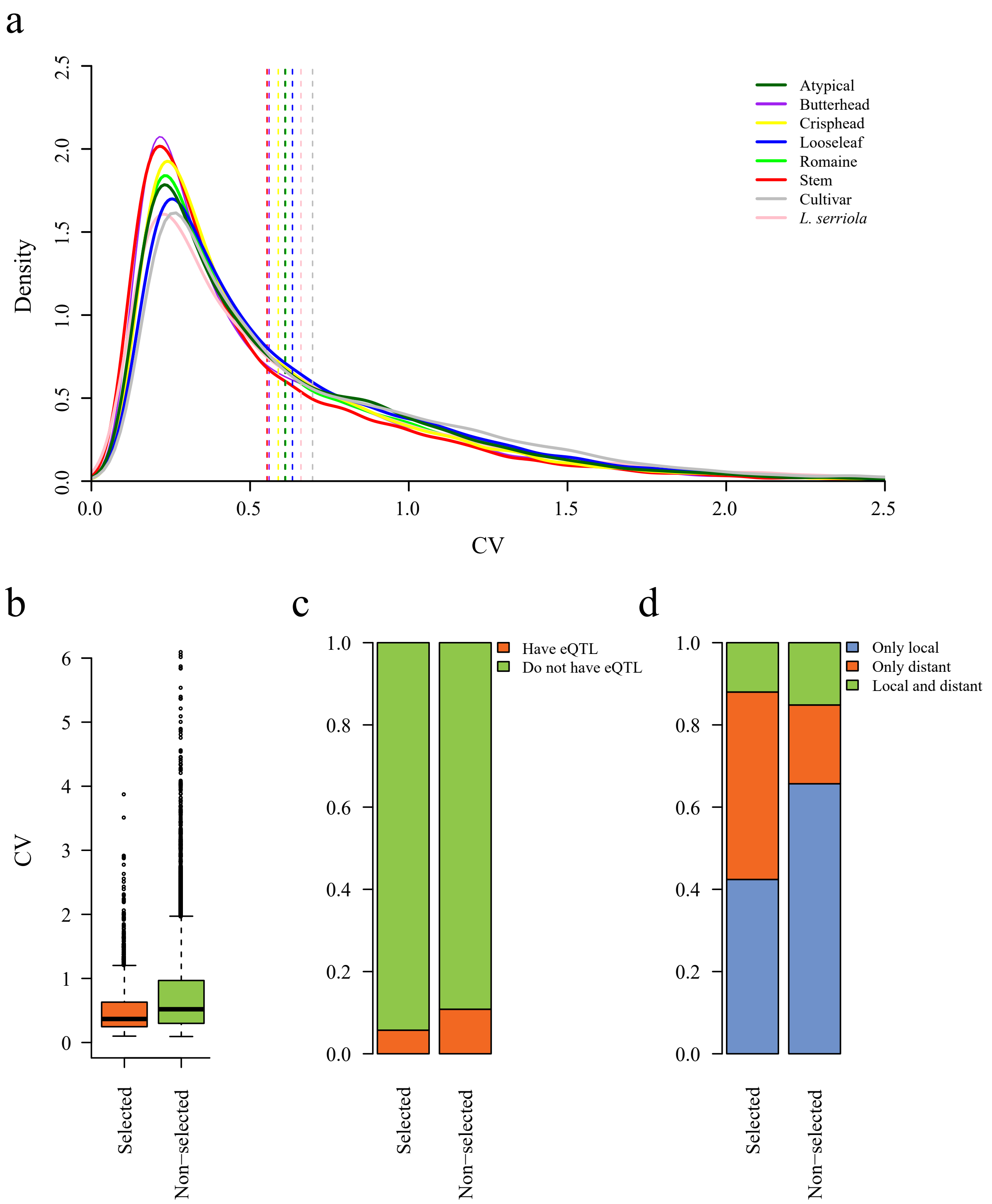

Supplementary Figure 12. Transcriptomic changes during domestication

(a) Density plots for the CV of gene expression in different groups. The dashed lines represent the mean value of the CV in a particular group. (b) Comparison of CV of gene expression between selected and non-selected genes in cultivated lettuce. (c) Distribution of eQTL in selected and non-selected genes. (d) Types of eQTL in selected and non-selected genes.

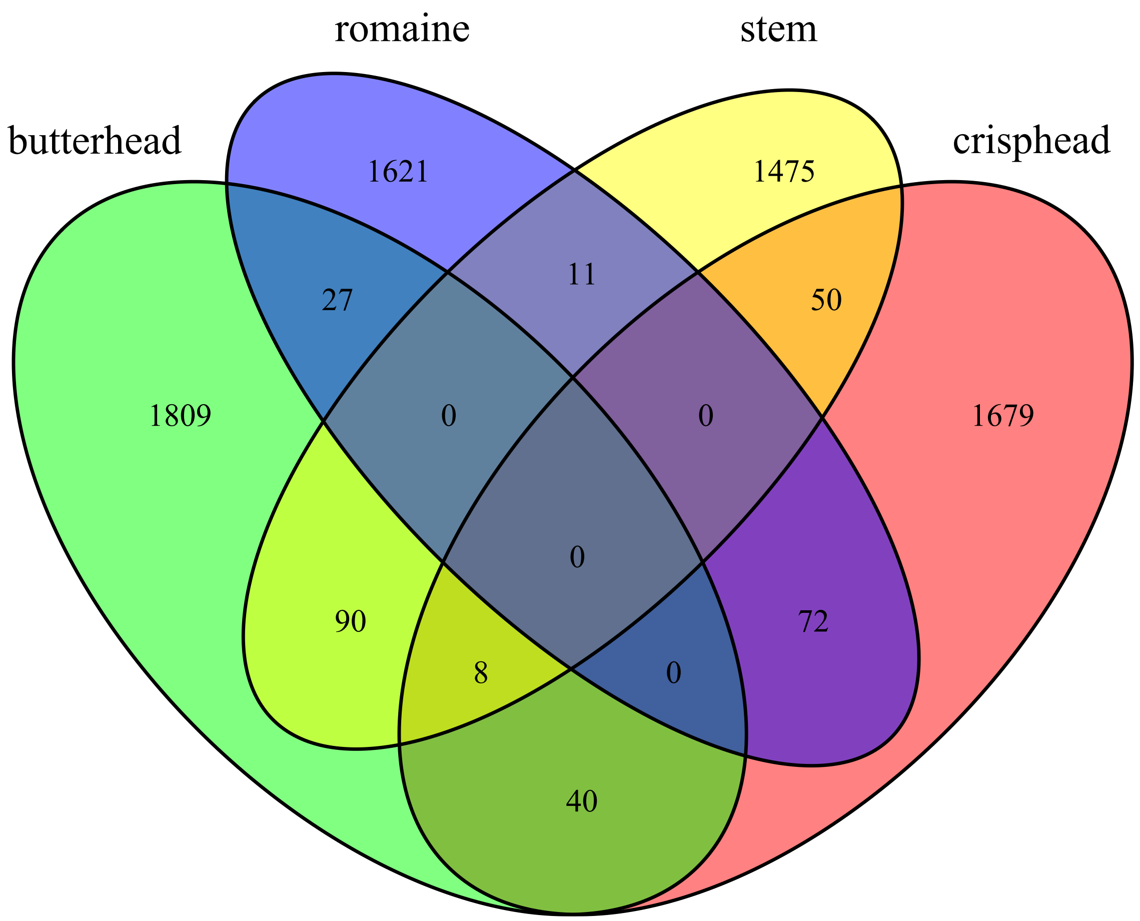

Supplementary Figure 13. Venn diagram of differentially selected genes identified in the four horticultural types

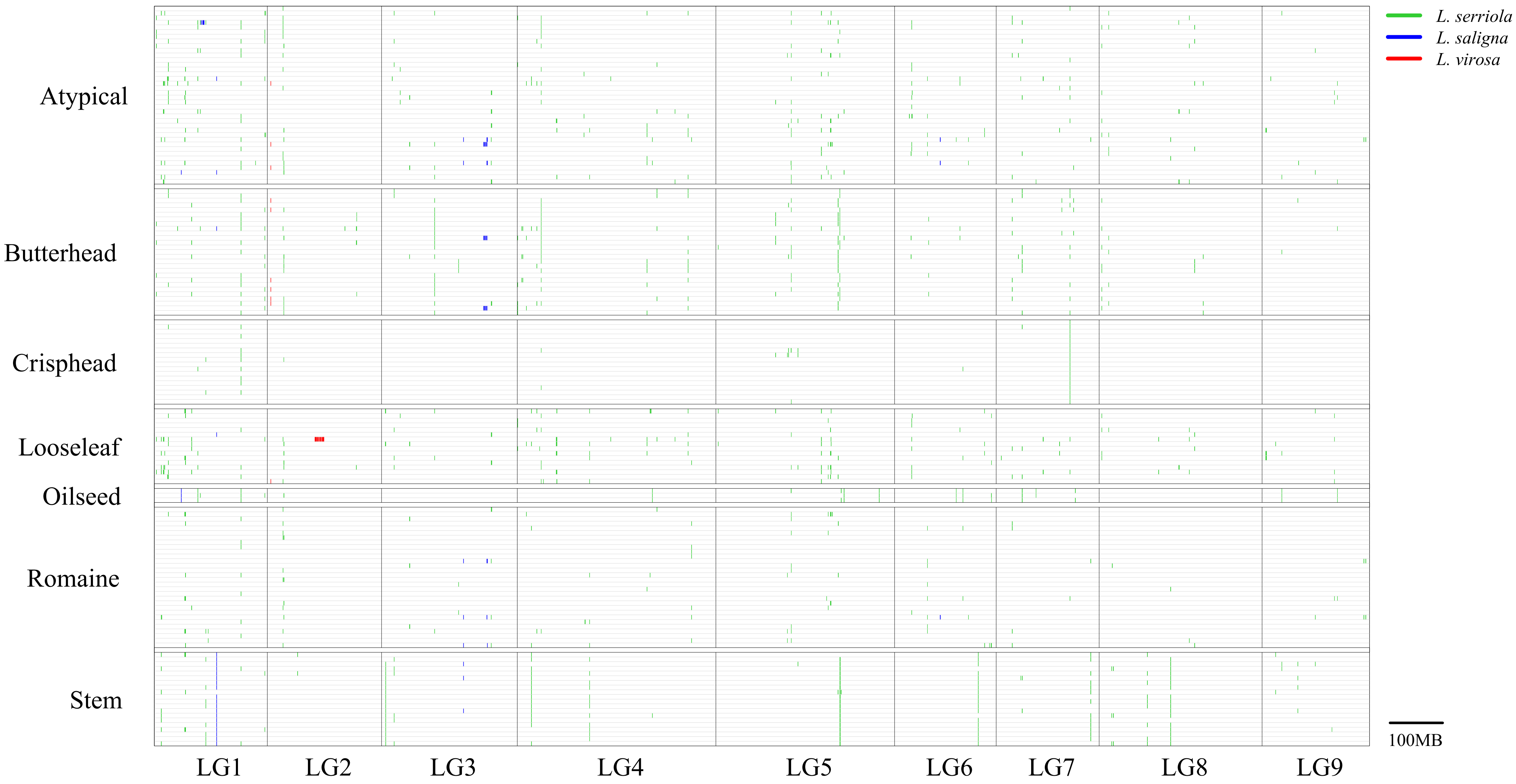

Supplementary Figure 14. Genome-wide analysis of introgressions from wild *Lactuca* species in cultivated lettuce

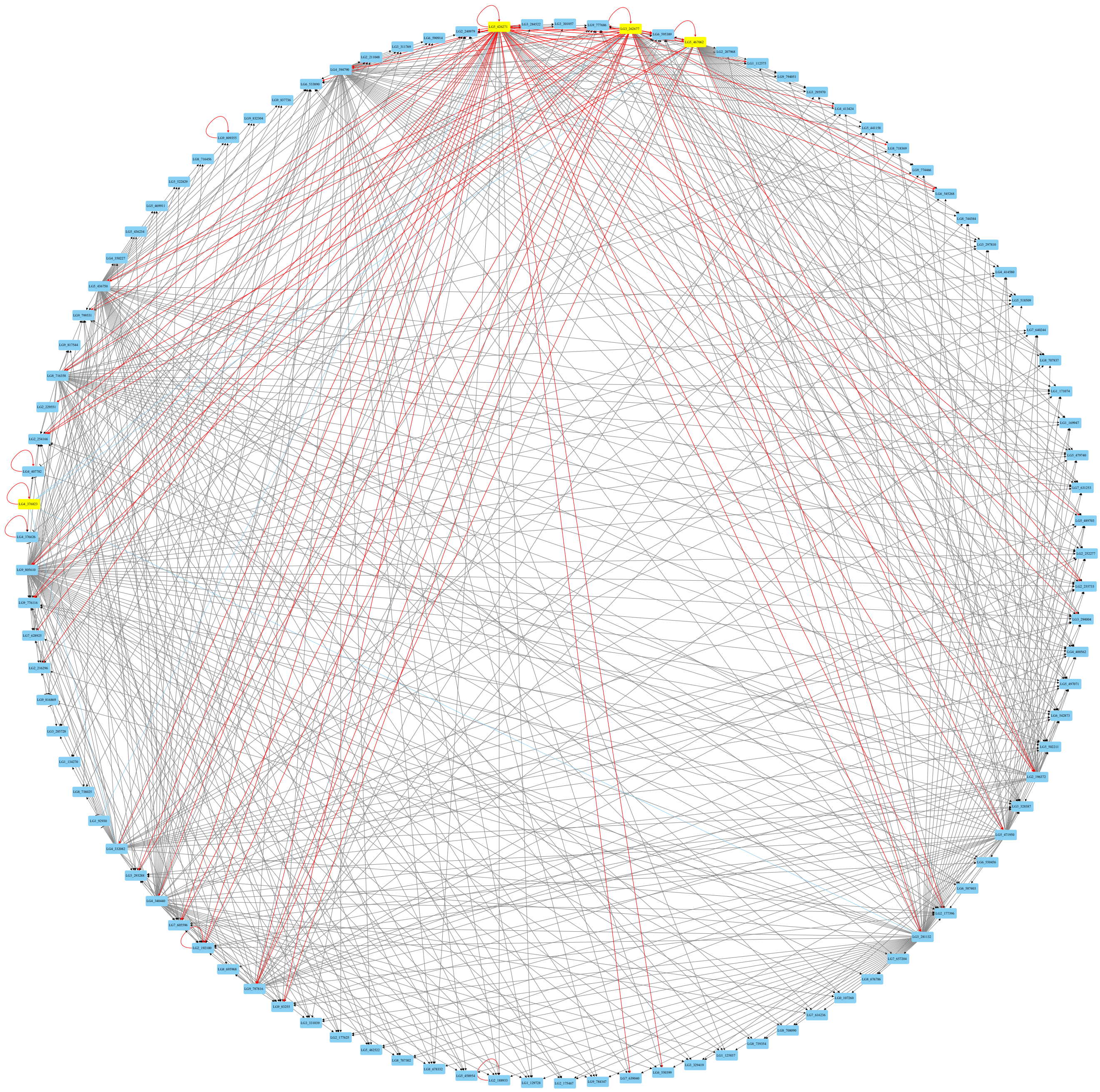

Supplementary Figure 15. Inferred regulatory network of genes involved in flavonoid biosynthesis

The regulatory network for flavonoid biosynthesis was inferred using the iGA and co-expression data. Each node is a gene. Each edge is a predicted regulatory connection between genes. Yellow nodes represent the identified four candidate regulatory genes. Blue nodes represent the target genes or other possible genes identified by co-expression analysis. Red arrows and blue bars have evidence of eQTL support, which represent as positive and negative regulation, respectively. Gray arrows are supported only by co-expression analysis.

a

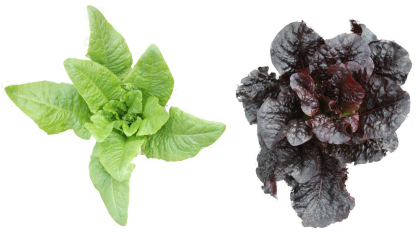

b

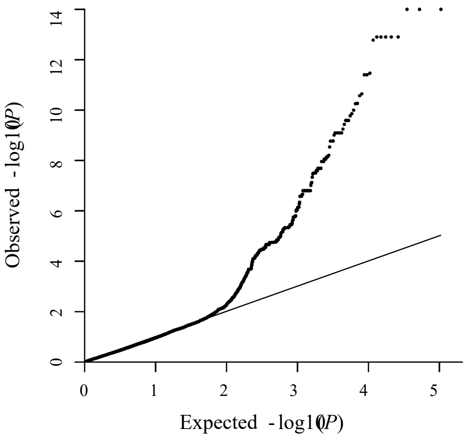

Supplementary Figure 16. Phenotype of leaf color and QQ plots

(a) Representative phenotypes of leaf color in associated population. (b) QQ plots of *P*-values for all SNPs.

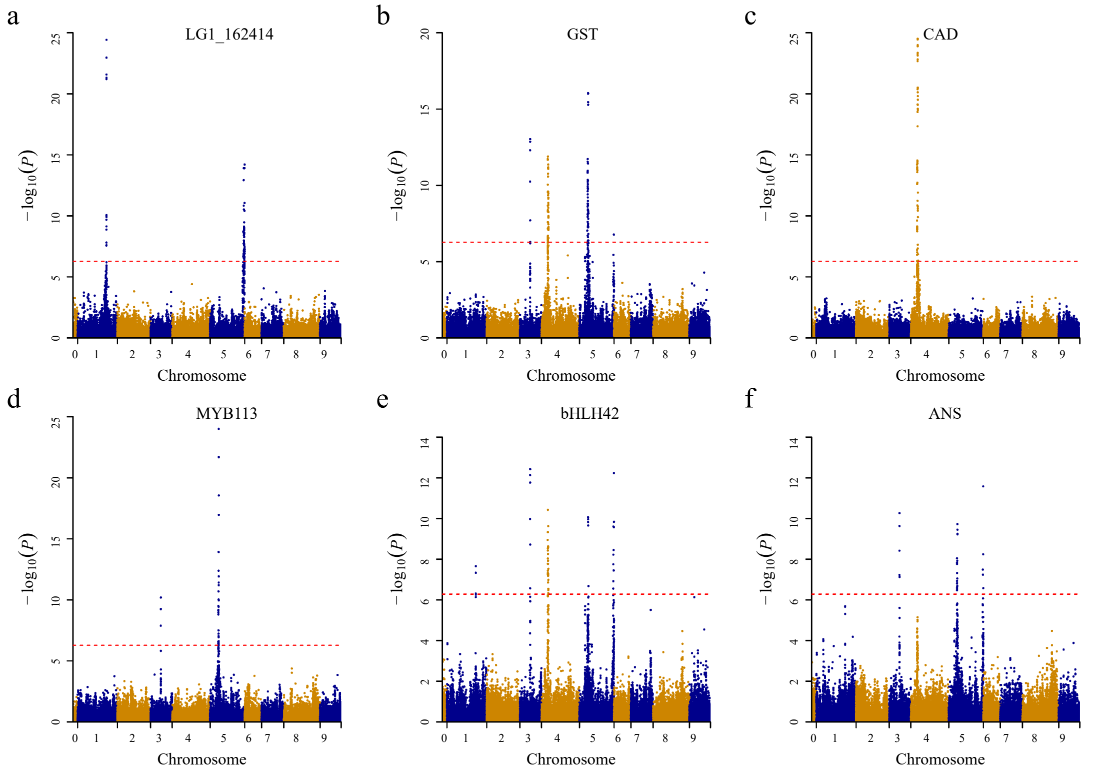

Supplementary Figure 17. The eQTL mapping results of the six candidate genes associated with leaf color in lettuce

(a) Manhattan plot from eQTL mapping of the first locus *LG1_162414*. One local eQTL and two distant eQTLs were detected for this gene. The distant eQTL on LG5 was coincident with the fifth locus. (b) Manhattan plot from the eQTL mapping of the second locus *GST* (LG3_262677). Three eQTLs were detected for this gene, including one local eQTL and two distant eQTLs. The two distant eQTLs were linked with the third and fourth locus, respectively. (c) Manhattan plot from the eQTL mapping of the third locus *CAD* (LG4_376823). Only one local eQTL was identified for this gene. (d) Manhattan plot from the eQTL mapping of the fourth locus *MYB113* (LG5_426271). One local eQTL and one distant eQTL were detected for this gene. The distant eQTL on LG3 was linked to the second locus. (e) Manhattan plot from the eQTL mapping of the fifth locus *bHLH42* (LG5_467062). One local eQTL and four distant eQTLs were detected for this gene. The four distant eQTLs were coincident with the locations of the first to fourth locus, respectively. (f) Manhattan plot from the eQTL mapping of the sixth locus *ANS* (LG9_787816). Three distant eQTLs of this gene overlapped with the second, fourth, and fifth locus, respectively. The red horizontal dashed line indicates the Bonferroni-corrected significance threshold ($-\log_{10}(P) = 6.31$, $\alpha = 0.05$).

Supplementary Tables:

Supplementary Table 1. Summary of SNPs in wild and cultivated lettuce

Groups	Total SNPs[a]	Intergenic	Upstream[b]	CDS	Intron	Downstream[b]
Atypical	176,349	22,767	17,971	103,948	11,190	20,473
Butterhead	148,708	24,045	15,827	80,916	10,410	17,510
Crisphead	111,302	25,172	12,688	50,930	9,139	13,373
Looseleaf	157,258	22,802	16,620	88,920	10,671	18,245
RIL	119,097	24,067	13,676	55,303	9,956	15,095
Romaine	141,168	22,191	15,087	77,649	9,490	16,751
Stem	122,263	24,053	13,654	59,721	9,664	15,171
Oilseed	133,171	29,462	15,673	56,680	13,316	18,040
Cultivar	220,383	23,088	22,231	134,347	14,361	26,356
Intermediate	175,206	26,442	18,644	95,723	13,460	20,937
L. serriola	305,232	27,139	29,779	195,615	17,021	35,678
L. saligna	270,824	36,755	31,080	138,329	24,196	40,484
L. virosa	369,501	33,702	36,386	232,768	22,976	43,669
Total	1,133,865	60,696	105,650	810,310	44,288	112,921

[a] SNPs were summarized before imputation.

[b] Upstream refers to a region that is within 3-kb upstream of the start codon. Downstream refers to a region that is within 3-kb downstream of the stop codon.

Supplementary Table 2. GO (biological process) enrichment analysis of genes that were highly impacted by SNPs

GO id	GO description	Query item	Reference item	Enrichment fold [a]	*P*-value
GO:0009595	detection of biotic stimulus	15	50	5.13	1.90E-06
GO:0006468	protein phosphorylation	152	1891	1.37	9.60E-05
GO:0052544	defense response by callose deposition in cell wall	12	55	3.73	2.70E-04
GO:0051606	detection of stimulus	24	177	2.32	3.20E-04
GO:0061077	chaperone-mediated protein folding	11	58	3.24	1.30E-03
GO:0006952	defense response	167	2332	1.22	5.40E-03
GO:0009626	plant-type hypersensitive response	36	386	1.59	6.80E-03
GO:0009788	negative regulation of abscisic acid-activated signaling pathway	14	109	2.20	8.10E-03
GO:0033554	cellular response to stress	118	1604	1.26	8.80E-03
GO:0008037	cell recognition	17	172	1.69	3.30E-02
GO:0010648	negative regulation of cell communication	21	226	1.59	3.40E-02
GO:0043269	regulation of ion transport	15	110	2.33	3.80E-03
GO:0040029	regulation of gene expression, epigenetic	24	263	1.56	2.90E-02

[a] Fold enrichment was calculated based on GO-annotated genes (1536) in the query list of genes (2035) per GO-annotated genes (26,264) in the reference genome list (38,915).

Supplementary Table 3. Pfam domain enrichment analysis of genes that were highly impacted by SNPs

Pfam id	Pfam description	Query item	Reference item	Enrichment fold [a]	*P*-value
PF00931	NB-ARC domain	47	367	2.23	2.35E-07
PF08263	Leucine rich repeat N-terminal domain	38	309	2.14	8.50E-06
PF13855	Leucine-rich repeat	45	423	1.85	5.43E-05
PF00069	Protein kinase domain	84	983	1.49	1.80E-04
PF01582	TIR domain	26	218	2.08	3.46E-04
PF07714	Protein tyrosine kinase	59	656	1.56	4.59E-04
PF01453	D-mannose binding lectin	18	144	2.17	1.57E-03
PF00954	S-locus glycoprotein domain	14	112	2.17	4.95E-03
PF12796	Ankyrin repeats (3 copies)	16	141	1.97	7.16E-03
PF08276	PAN-like domain	11	91	2.10	1.51E-02
PF13812	Pentatricopeptide repeat domain	6	40	2.61	2.56E-02
PF00564	PB1 domain	7	51	2.39	2.58E-02
PF00149	Calcineurin-like phosphoesterase	9	75	2.09	2.77E-02
PF00664	ABC transporter transmembrane region	7	55	2.21	3.72E-02
PF00201	UDP-glucoronosyl and UDP-glucosyl transferase	17	187	1.58	4.16E-02
PF13499	EF-hand domain pair	12	122	1.71	4.81E-02
PF00931	NB-ARC domain	47	367	2.23	2.35E-07
PF08263	Leucine rich repeat N-terminal domain	38	309	2.14	8.50E-06

[a] Fold enrichment was calculated based on Pfam-annotated genes (1422) in the query list of genes (2035) per Pfam-annotated genes (24,742) in the reference genome list (38,915).

Supplementary Table 4. Pairwise comparisons of *F*st values between different groups/types

	Atypical	Butterhead	Crisphead	Romaine	Stem	Looseleaf	Cultivar	*L. serriola*
**Atypical**	-	-	-	-	-	-	-	-
**Butterhead**	0.12	-	-	-	-	-	-	-
**Crisphead**	0.15	0.36	-	-	-	-	-	-
**Romaine**	0.11	0.28	0.32	-	-	-	-	-
**Stem**	0.36	0.47	0.57	0.46	-	-	-	-
**Looseleaf**	0.02	0.15	0.24	0.16	0.39	-	-	-
**Cultivar**	0.01	0.10	0.13	0.08	0.26	0.03	-	-
L. serriola	0.50	0.52	0.54	0.54	0.54	0.45	0.56	-

Supplementary Table 5. Support for the one-population models defined in Supplementary Fig. 8

Groups	Model	Number of runs	Number of parameters	Log likelihood (Lhood)	AIC	ΔAIC	Akaike weights (w_i)
Butterhead	model 5	50	5	-46186.25	212705.54	0.00	0.44
	model 4	25	4	-46186.96	212706.82	1.29	0.23
	model 2	25	3	-46187.66	212708.02	2.48	0.13
	model 6	25	5	-46186.86	212708.33	2.80	0.11
	model 3	25	3	-46187.83	212708.81	3.27	0.09
	model 1	25	1	-46323.11	213327.80	622.26	0.00
Crisphead	model 5	25	5	-24867.11	114527.28	0.00	1.00
	model 4	25	4	-24872.02	114547.90	20.63	0.00
	model 6	25	5	-24872.08	114550.16	22.88	0.00
	model 3	25	3	-24896.53	114658.74	131.47	0.00
	model 2	25	3	-24896.73	114659.68	132.40	0.00
	model 1	25	1	-24902.79	114683.59	156.31	0.00
Romaine	model 5	50	5	-42722.92	196756.29	0.00	0.61
	model 4	25	4	-42723.61	196757.51	1.22	0.33
	model 6	25	5	-42723.99	196761.24	4.95	0.05
	model 2	25	3	-42737.52	196819.54	63.25	0.00
	model 3	25	3	-42740.74	196834.40	78.11	0.00
	model 1	25	1	-42745.28	196851.29	95.00	0.00
Stem	model 5	50	5	-38612.34	177826.38	0.00	0.31
	model 2	25	3	-38613.24	177826.56	0.18	0.28
	model 3	25	3	-38613.33	177826.95	0.57	0.23
	model 6	25	5	-38612.87	177828.85	2.47	0.09
	model 4	25	4	-38613.32	177828.91	2.53	0.09
	model 1	25	1	-38632.07	177909.26	82.88	0.00
L. serriola	model 5	25	5	-126189.86	581135.80	0.00	0.87
	model 2	25	3	-126191.61	581139.83	4.03	0.12
	model 3	25	3	-126192.59	581144.33	8.53	0.01
	model 6	25	5	-126192.81	581149.36	13.56	0.00
	model 4	25	4	-126193.43	581150.20	14.40	0.00
	model 1	25	1	-126564.43	582852.72	1716.92	0.00

Supplementary Table 6. Support for the two-population models defined in Supplementary Fig. 9

Groups	Model	Number of runs	Number of parameters	Log likelihood (Lhood)	AIC	ΔAIC	Akaike weights (w_i)
Butterhead	model 1	25	9	-169125.49	778869.64	35.99	≈ 0
	model 2	25	13	-169115.93	778833.65	0.00	≈ 1
Crisphead	model 1	25	9	-156326.14	719926.50	44.88	≈ 0
	model 2	25	13	-156314.66	719881.62	0.00	≈ 1
Romaine	model 1	25	9	-168468.61	775844.6	173.49	≈ 0
	model 2	25	13	-168429.2	775671.12	0	≈ 1
Stem	model 1	25	9	-166437.16	766489.42	397.73	≈ 0
	model 2	25	13	-166349.05	766091.69	0	≈ 1

Supplementary Table 7. Support for the four-population models defined in Supplementary Fig. 10

Model	Number of runs	Number of parameters	Log likelihood (Lhood)	AIC	ΔAIC	Akaike weights (w_i)
model 1	50	17	-154038.85	709409.11	1471.95	≈ 0
model 2	50	17	-154375.83	710960.98	3023.82	≈ 0
model 3	50	17	-154060.31	709507.96	1570.80	≈ 0
model 4	50	17	-154122.00	709792.05	1854.89	≈ 0
model 5	50	21	-153717.48	707937.16	0.00	≈ 1
model 6	50	21	-154080.84	709610.50	1673.34	≈ 0
model 7	50	23	-154956.98	713649.27	5712.11	≈ 0
model 8	50	22	-153742.92	708056.30	119.14	≈ 0
model 9	50	22	-154053.49	709486.55	1549.39	≈ 0
model 10	50	22	-154681.19	712377.22	4440.06	≈ 0
model 11	50	21	-153836.62	708485.83	548.66	≈ 0
model 12	50	23	-154116.03	709776.54	1839.38	≈ 0

Supplementary Table 8. Support for the five-population models defined in Figure 2.a and Supplementary Fig. 11

Model	Number of runs	Number of parameters	Log likelihood (Lhood)	AIC	ΔAIC	Akaike weights (w_i)
single-founder model	100	27	-153284.11	705953.41	0	≈ 1
double-founder model	100	31	-155209.30	714827.25	8873.84	≈ 0

Supplementary Table 9. Parameter estimates and confidence intervals for the lettuce demographic model defined in Fig. 2a

Parameters	Description	Point estimate	95% CI[a]
Nanc	Ancestral effective population size	89,744	76,475-108,348
Nbw	Effective population size of the *L. serriola* population during the founder bottleneck	10,045	5,739-14,319
Ncw	Effective population size of the *L. serriola* population	156,874	115,853-179,709
Nba	Effective population size of the ancestral cultivated lettuce population during the founder bottleneck	8,295	3,083-12,298
Nca	Effective population size of the ancestral cultivated lettuce population	10,374	9,847-11,599
Nbs	Effective population size of the stem lettuce population during the founder bottleneck	1,516	972-2,435
Ncs	Effective population size of the stem lettuce population	11,552	1-29,830
Nbr	Effective population size of the romaine population during the founder bottleneck	456	139-679
Ncr	Effective population size of the romaine population	47,238	10,324-92,491
Nbc	Effective population size of the crisphead population during the founder bottleneck	217	53-283
Ncc	Effective population size of the crisphead population	22,884	451-80,983
Nbb	Effective population size of the butterhead population during the founder bottleneck	352	192-994
Ncb	Effective population size of the butterhead population	56,235	12,116-90,532
Tsw	Time that the *L. serriola* founder bottleneck began	12,804	11,483-15,756
Tew	Time that the *L. serriola* founder bottleneck ended	8,132	7,779-10,216
Tsa	Divergence time between *L. serriola* and the ancestral cultivated lettuce population	10,829	10,391-13,005
Tea	Time that the ancestral cultivated lettuce founder bottleneck ended	8,359	5,584-12,061
Tss	Time that the stem lettuce founder bottleneck began	1,922	1,730-3,036
Tes	Time that the stem lettuce founder bottleneck ended	776	502-1,194
Tsr	Time that the romaine founder bottleneck began	456	303-636
Ter	Time that the romaine founder bottleneck ended	197	92-269
Tsc	Time that the crisphead founder bottleneck began	527	272-691
Tec	Time that the crisphead founder bottleneck ended	131	86-212
Tsb	Time that the butterhead founder bottleneck began	296	246-687
Teb	Time that the butterhead founder bottleneck ended	132	96-240
MRWC	Migration rates between *L. serriola* and four horticultural types each	5.49×10^{-5}	3.30×10^{-5}-7.55×10^{-5}
MRHT	Migration rates between different horticultural types	7.66×10^{-5}	5.12×10^{-5}-1.75×10^{-4}

[a] 95% confidence intervals were obtained by bootstrapping 4DTV sites and performing parameter inference on each bootstrap dataset with 50 runs of *fastsimcoal2*.

Supplementary Table 10. Genome-wide nucleotide diversity (π) and expression diversity (CV) for each group

Group	π	L_{π}[a]	CV	L_{CV}[b]
Atypical	2.04E-03	57.78%	0.61	7.63%
Butterhead	1.68E-03	65.19%	0.56	15.22%
Crisphead	8.94E-04	81.52%	0.59	10.84%
Romaine	1.45E-03	70.09%	0.61	7.32%
Stem	1.30E-03	73.03%	0.56	15.22%
Looseleaf	2.11E-03	56.38%	0.63	4.06%
Cultivar	2.14E-03	55.83%	0.70	-5.53%
L. serriola	4.84E-03	-	0.66	-

[a]L_{π}, loss of nucleotide diversity, calculated as $L_{\pi} = 1 - (Group_{\pi}/Serriola_{\pi})$

[b]L_{CV}, loss of expression diversity, calculated as $L_{CV} = 1 - (Group_{CV}/Serriola_{CV})$

Supplementary Table 11. GO (biological process) enrichment analysis of genes that were selected in cultivated lettuce

GO id	GO description	Query item	Reference item	Enrichment fold [a]	*P*-value
GO:0090487	secondary metabolite catabolic process	12	30	6.13	5.30E-06
GO:0009404	toxin metabolic process	14	44	4.88	7.80E-06
GO:0006790	sulfur compound metabolic process	47	386	1.87	0.00011
GO:0044248	cellular catabolic process	134	1606	1.28	0.0034
GO:0051188	cofactor biosynthetic process	35	329	1.63	0.0057
GO:0006996	organelle organization	177	2232	1.22	0.0058
GO:0048767	root hair elongation	10	56	2.74	0.0066
GO:0009695	jasmonic acid biosynthetic process	7	33	3.25	0.01
GO:0048468	cell development	40	411	1.49	0.012
GO:0007623	circadian rhythm	20	174	1.76	0.016
GO:0048511	rhythmic process	22	200	1.69	0.018
GO:0019685	photosynthesis, dark reaction	7	39	2.75	0.021
GO:0033013	tetrapyrrole metabolic process	17	145	1.80	0.021
GO:0007033	vacuole organization	13	106	1.88	0.03
GO:0009611	response to wounding	24	239	1.54	0.034
GO:0016116	carotenoid metabolic process	6	34	2.71	0.034
GO:0009914	hormone transport	15	132	1.74	0.036
GO:0023056	positive regulation of signaling	13	110	1.81	0.038

[a] Fold enrichment was calculated based on GO-annotated genes (1713) in the query list of genes (2178) per GO-annotated genes (26,264) in the reference genome list (38,915).

Supplementary Table 12. List of group-specific introgressed regions

Chromosome	Start	End	Horticultural type that possessed	Source of the introgressed region
LG1	118280001	118660000	Stem	*L. saligna*
LG3	7680001	8060000	Stem	*L. serriola*
LG4	26720001	27220000	Stem	*L. serriola*
LG5	235540001	236540000	Stem	*L. serriola*
LG6	158840001	159220000	Stem	*L. serriola*
LG7	140100001	140540000	Crisphead	*L. serriola*
LG8	135780001	136160000	Stem	*L. serriola*

Supplementary Table 13. Significant regulators of flavonoid biosynthesis identified using the iGA approach

Group number	Regulator	Gene annotation of the regulator	*P*-Value Changed[a]	Targets	PCC[b]	Annotation of the target
1	LG3_262677	Glutathione *S*-transferase (GST, TT19)	1.65×10^{-10}	LG2_196372	0.93	Dihydroflavonol reductase (DFR)
				LG3_281132	0.78	Flavanone 3-hydroxylase (F3H)
				LG4_394790	0.76	bHLH transcription factor Glabra 2 (GL2)
				LG5_426271	0.73	MYB-type transcription factor (MYB113)
				LG5_436750	0.43	4-coumarate:CoA ligase1 (4CL1)
				LG5_467062	0.88	bHLH transcription factor 42 (bHLH42, TT8)
				LG5_471950	0.67	Cytochrome P450 75B1 (CYP75B1, TT7)
				LG8_716358	0.70	Chalcone-flavanone isomerase (CHI)
				LG9_787816	0.84	Leucoanthocyanidin dioxygenase (ANS, TT18)
				LG9_805610	0.66	Chalcone isomerase (CHI)
2	LG5_426271	MYB-type transcription factor (MYB113)	3.13×10^{-10}	LG2_196372	0.68	Dihydroflavonol reductase (DFR)
				LG3_262677	0.73	Glutathione *S*-transferase (GST, TT19)
				LG3_281132	0.69	Flavanone 3-hydroxylase (F3H)
				LG4_332082	0.75	R3-type MYB transcription factor (CPC)
				LG4_340440	0.45	Anthocyaninless 2 (ANL2)
				LG4_394790	0.80	bHLH transcription factor Glabra 2 (GL2)
				LG5_436750	0.57	4-coumarate:CoA ligase1 (4CL1)
				LG5_467062	0.73	bHLH transcription factor 42 (bHLH42, TT8)
				LG5_471950	0.56	Cytochrome P450 75B1 (CYP75B1, TT7)
				LG8_716358	0.62	Chalcone-flavanone isomerase (CHI)
				LG9_787816	0.67	Leucoanthocyanidin dioxygenase (ANS, TT18)
				LG9_805610	0.63	Chalcone isomerase (CHI)
3	LG4_376823	Cinnamyl-alcohol dehydrogenase (CAD)	6.22×10^{-9}	LG3_262677	-0.45	Glutathione *S*-transferase (GST, TT19)
				LG3_281132	-0.39	Flavanone 3-hydroxylase (F3H)
				LG4_332082	-0.52	R3-type MYB transcription factor (CPC)
				LG5_467062	-0.45	bHLH transcription factor 42 (bHLH42, TT8)
4	LG5_467062	bHLH transcription factor (TT8)	4.35×10^{-7}	LG2_196372	0.89	Dihydroflavonol reductase (DFR)
				LG2_229551	0.53	Chalcone synthase (CHS)
				LG9_787816	0.84	Leucoanthocyanidin dioxygenase (ANS, TT18)

[a] The threshold for the *P*-value was 0.01 / (total number of putative regulators) = 1.47×10^{-5}, where the total number of putative regulators was 681.

[b] PCC between the target genes and candidate master regulators.

Supplementary Table 14. CAPs markers used for linkage analysis in this study

Name	SNP Position	Primer sequences (5'-3')	Enzyme	Purpose
M3_left	LG4: 47920680	CCTCCTCATAAAGCTCGGGTGCC	EcoRI	linkage analysis of locus 3
		TCGGGAAGTTCTCGGTAAAGGTGC		
M3_right	LG4: 53226729	GCCAGATTCAAGTCACGTACACAGA	MboI	
		ACGTGCAAGACACGTGGGGG		
M4_left	LG5: 84600044	TCACCCCATTGTAACTTTGTGT	HindIII	linkage analysis of locus 4
		ACACAATCCACAGAGAGCGT		
M4_right	LG5: 86116763	TGACCCCCAAAATACCTGCA	EcoRI	
		AAGGTGAGCAGCCACATCAA		
M5_left	LG5: 334839952	AAGGAGGCGGTCAAGTCATG	EcoRI	linkage analysis of locus 5
		ATGGACCATTTAACCCGCGT		
M5_right	LG5: 337620880	TCCTTGATTACTCGTCCCCA	BstBI	
		GCTCGATGCTCTATGGGTATGT		
M6_left	LG5: 151249579	GCCAAGGCAATGGAATTGGT	XbaI	linkage analysis of locus 6
		CTGCCGTACCGACACACTAT		
M6_right	LG9: 156891057	TAAATGTCGTGGTTGGCCCA	XbaI	
		ACGTCATCATGCCACGTCAG		

Supplementary Notes:

Supplementary Note 1. Large-effect SNPs found in 2035 genes

A total of 810,310 SNPs (71.46%) were mapped to the coding regions (CDS) of 24,042 genes **(Supplementary Table 1)**. The potential effects of SNPs in CDS were investigated using SnpEff[2]. A total of 540,196 SNPs in the CDS regions were found to be synonymous and 270,114 SNPs were nonsynonymous. Interestingly, 2346 SNPs (large-effect SNPs) from 2035 genes caused premature stop codons, modified start or stop codons, or induced disruptive splice variants, etc. These large-effect SNPs probably abolish or dramatically change the functions of the affected genes, including some well-studied genes, such as *LD* (luminidependens, LG8_748409)[3] and *NPR3* (nonexpresser of PR genes 3, LG8_739485)[4] **(Supplementary Data 2)**. GO and protein domain enrichment analysis were used to further assess functional features of these affected genes. GO analysis detected enrichment in biological processes such as defense response and protein phosphorylation **(Supplementary Table 2)**, and Pfam analysis revealed that genes encoding disease-related proteins and kinase were significantly overrepresented **(Supplementary Table 3)**, which were consistent with previous findings[5,6].

Supplementary Note 2. Imputation of missing genotypes

The fillGenotype software[7] was used to impute the missing data for further analysis. The accessions of *L. saligna* and *L. virosa* were excluded from imputation because they exhibited many species' specific SNPs that are absent in either *L. sativa* or *L. serriola*. Consequently, 217 accessions were used to perform SNP imputation. By randomly masking 1% of the SNP sites, a simulation was performed to determine the imputation accuracy and the filling rate **(Supplementary Fig. 5)**. The optimal imputation accuracy (97.95%) and filling rate (97.90%) were achieved when the missing data rate cutoff value was set at 0.8 and the following values were used for the fillGenotype parameters: w = 20, p = -11, k = 9 and r = 0.5. A total of 344,222 SNPs

with missing rates ≤ 0.8 were filled using this imputation method.

Supplementary Note 3. Demographic model selection for one-, two- and four-population models

We tested one-, two- and four-population models to help guide the development of demographic models for lettuce. For the one-population model, six different demographic models were tested **(Supplementary Fig. 8)**. A three-epoch model with a population bottleneck of some duration followed by a recovery **(model 5 in Supplementary Fig. 8)** was the best-fitting model for all five populations **(Supplementary Table 5)**. Based on these results, we suggest that all populations have experienced bottlenecks in their history.

In the two-population models, each horticultural type was combined with *L. serriola* as the source population **(Supplementary Fig. 9)**. Model 2 had the best support for all horticultural types **(**$w_i \approx 1$**, Supplementary Table 6)**. This model is consistent with the idea that there were two evolutionary stages for each horticultural type, the domestication stage (leading to primitive cultivated lettuce) and the improvement stage (leading to modern cultivated lettuce).

Then, four-population models were analyzed for *L. serriola* and three leafy horticultural types (butterhead, crisphead and romaine). Stem lettuce was excluded from the models, based on the finding that it is genetically the most distinct group within cultivated lettuce. Twelve models **(Supplementary Fig. 10)** were tested for different topologies within three horticultural types, and model 5 provided the best support for the data **(**$w_i \approx 1$**, Supplementary Table 7)**. This model suggested that these three horticultural types developed independently from an ancestral cultivated population.

Supplementary Note 4. Genes involved in stress, organ development and metabolic processes are selected during domestication

Jasmonic acid (JA) is a natural hormone that plays a central role in plant responses

to biotic and abiotic stresses[8]. For example, *4CL* (4-coumarate:CoA ligases, LG8_676500)[9], *ACX1* (Acyl-coa oxidase 1, LG6_543615)[10], *AOC* (Allene oxide cyclase, LG4_389845)[11], *JAI3* (Jasmonate-insensitive 3, LG8_696894)[12], *KAT2* (3-ketoacyl-coa thiolate 2, LG6_552932)[10] and *PLA1* (Phospholipase A1, LG7_633827)[13], which involved in metabolism of JA, were all found in regions under selection.

Compared with *L. serriola*, morphological features of cultivated lettuce have greatly changed, such as delayed bolting[14], larger seed size, and shallow root system with a short taproot and prolific lateral branches[15]. The time of bolting is an important agronomic trait in lettuce, because it affects the quality, yield and scheduling of production[16,17]. Bolting is a key transition from vegetative to reproductive (i.e., flowering) phase in lettuce. Genes associated with bolting or flowering were identified in the regions under selection, such as *AGL24* (Agamous-like 24, LG6_537816)[18], *GI* (Gigantea, LG6_598704)[19], *LHY* (Late elongated hypocotyl, LG3_287450)[20], *PRR7* (Pseudo-response regulator 7, LG8_758125)[21] and *RVE8* (Reveille 8, LG2_180610)[22]. Genes associated with seed and root development were also found in our analysis, including *TTG2* (Transparent testa glabra 2, LG2_180843) and *IKU1* (Haiku, LG6_578206), regulating endosperm growth and seed size[23,24]; *ARK1* (Armadillo-repeat kinesin1, LG8_733861) promoting microtubule catastrophe during root hair development[25]; *JAI1* (Jasmonate insensitive 1, LG6_589609), regulating root meristem activity and stem cell niche maintenance[26]; and *PLDZ1* (Phospholipase D zeta 1, LG6_576132), regulating root development in response to nutrient limitation[27].

Metabolic processes influencing nutrition and food functionality were also shown to be under selection during domestication. Lettuce is a source of vitamins (vitamin A, B1, B6, C, E, etc.) and minerals (potassium, etc.) for human[28]. Genes related to these metabolites were found under selection, such as *TPK1* (Thiamin pyrophosphokinase 1, LG9_786996)[29], *PDX1.2* (Pyridoxine biosynthesis 1.2, LG5_427483)[30], *VTC2* (Vitamin C defective 2, LG5_505590)[31], *VTE1* (Vitamin E deficient 1, LG5_500054)[32], *TPK1* (Two pore K^+ channel 1, LG6_534072)[33], *SOS1* (Salt overly sensitive 1, LG6_568389)[34], *SOS2* (Salt overly sensitive 2, LG7_654166)[34] and *SKOR* (Stelar K^+ outward rectifier, LG3_326118)[35].

Supplementary Note 5. Genes associated with leaf-heading and secondary stem growth are selected in crisphead and stem types, respectively

Leaf-heading and secondary stem growth are important traits in cultivated lettuce, particularly in crisphead and stem types, respectively. Leaves are derived from primordia on the flanks of shoot apical meristem (SAM) followed by asymmetric outgrowth, expansion and maturation[36]. Screening the selected genes in crisphead type, we identified several genes that may be associated with leaf-heading in crisphead type. In a recent study, it is revealed that genes involved in establishing adaxial-abaxial polarities of leaf primordia are responsible for leaf-heading in *Brassica rapa* and *Brassica oleracea*[37,38]. We speculated that the same molecular mechanism may also control leaf-heading phenotype in crisphead type. The homologue of *ATHB15* (*Arabidopsis thaliana* homeobox 15, LG1_164856) which is a member of the class III HD-ZIP protein family to specify abaxial cell fate[37] was found to be under selection. It is known that the genetic control system for leaf primordium-SAM boundary formation has an important function in genetic network for leaf polarities[39]. Genes associated with the polarity regulation network were selected in crisphead type, such as *BOP2* (Blade on petiole 2, LG5_523032), which positively regulates the expression of the *AS2* gene on the adaxial sides of leaf primordium bases[39]. Genes that are important to leaf development were also identified in our analysis, such as *CLV1* (Clavata 1, LG2_207770), specifying and maintaining shoot meristem identity[40]; and auxin influx carrier *AUX1* (Auxin resistant 1, LG5_482970), triggering development of leaf primordium via accumulation peaks of auxin at the flank of the SAM[40].

Secondary growth of stem involves the thickening of the plant axis through the activity of vascular cambium (i.e., secondary meristem), leading to increased amounts of vascular tissues. Several genes that are related to vascular development or cell wall organization are located in selected regions in stem lettuce. Genes that regulate vascular stem cells are found in selected regions, such as *ATH1* (Arabidopsis thaliana homeobox

gene 1, LG3_329662)[41], *TOAD2* (Toadstool 2, LG4_364824)[42] and *OPS* (Octopus, LG8_722826)[42]. Secondary cell wall is vital for plant growth, typically in stem tissue for protection, structural support, as well as water and nutrients transport[43]. Genes involved in secondary wall biosynthesis were also found to be under selection, including three cellulose synthase genes: *CESA1* (Cellulose synthase 1, LG5_453514)[44], *CSLA2* (Cellulose synthase-like A2, LG6_568914)[43] and *IRX6* (Irregular xylem 6, LG4_374430)[43]; two xylan biosynthetic genes: *IRX14* (Irregular xylem 14, LG2_209670)[43] and *GLZ1* (Gaolaozhuangren 1, LG4_406605)[43]; and one gene required for lignin biosynthesis: *C4H* (Cinnamate 4-hydroxylase, LG8_701431)[43].

Supplementary Note 6. Linkage analysis of four loci associated with leaf color in lettuce

Six loci were identified to control color variation of lettuce using GWAS in our study. Sometimes it is challenging to construct a population that only one of the color controlling genes is segregating. Using different crosses and sub-populations, we succeeded in analyzing four loci (locus 3 to 6 in our study) that control leaf color in lettuce. These four loci are described as follows:

Linkage analysis of locus 3

A sub-population comprising 173 individuals was found showing a segregating ratio of green (130) : red (43) = 3 : 1 (χ^2 test, P = 0.9650, χ^2 = 0.002). Further analysis showed that the trait is controlled by locus 3. Two CAPs markers from the candidate region were designed **(Supplementary Table 14)**. Screening the segregating population showed that they co-segregated with leaf color in this population, confirming our GWAS results.

Linkage analysis of locus 4

One segregating population with 163 individuals derived from Multi-parent Advanced Generation Intercrosses (MAGIC) population was found. Among these individuals, 122 had red leaves and 41 had green leaves. χ^2 test showed that the trait is

controlled by a single gene (P = 0.9639, χ^2 = 0.002). Further analysis showed that the trait is controlled by locus 4. Two CAPs markers flanking the candidate gene were designed **(Supplementary Table 14)**. These two markers co-segregated with leaf color in this population, confirming our GWAS results.

Linkage analysis of locus 5

A $F_{2:3}$ sub-population (n = 167) which was derived from a cross between PI344074 and PI536839 was found with segregation of leaf color. A ratio of red (128) : green (39) ≈ 3 : 1 (χ^2 test, P = 0.6231, χ^2 = 0.242) individuals were found in this population, suggesting a single gene controlling this trait. Further analysis showed that the trait is controlled by locus 5. Two CAPs markers flanking the candidate gene were developed **(Supplementary Table 14)**. These two markers co-segregated with leaf color in this population, confirming our GWAS results.

Linkage analysis of locus 6

Another segregating population comprising 157 $F_{2:3}$ individuals derived from a cross between PI491070 and PI536760 was found. There were 126 and 31 individuals with red and green leaves, respectively. χ^2 test showed that the trait is controlled by a single gene (P = 0.1284, χ^2 = 2.312). Further analysis showed that the trait is controlled by locus 6. Two CAPs markers flanking the candidate gene were designed **(Supplementary Table 14)**. These two markers co-segregated with leaf color in this population, confirming our GWAS results.

Supplementary References

1. Kilibarda, M. & Bajat, B. PlotGoogleMaps : The R-Based Web-Mapping Tool for Thematic Spatial Data. *Geomatica* **66**, 37-49 (2012).
2. Cingolani, P. *et al.* A program for annotating and predicting the effects of single nucleotide polymorphisms, SnpEff: SNPs in the genome of Drosophila melanogaster strain w1118; iso-2; iso-3. *Fly (Austin)* **6**, 80-92 (2012).
3. Lee, I. *et al.* Isolation of LUMINIDEPENDENS: a gene involved in the control of flowering time in Arabidopsis. *Plant Cell* **6**, 75-83 (1994).
4. Fu, Z.Q. *et al.* NPR3 and NPR4 are receptors for the immune signal salicylic acid in plants. *Nature* **486**, 228-32 (2012).
5. Clark, R.M. *et al.* Common sequence polymorphisms shaping genetic diversity in Arabidopsis thaliana. *Science* **317**, 338-42 (2007).
6. Lai, J. *et al.* Genome-wide patterns of genetic variation among elite maize inbred lines. *Nat Genet* **42**, 1027-30 (2010).
7. Huang, X. *et al.* Genome-wide association studies of 14 agronomic traits in rice landraces. *Nat Genet* **42**, 961-7 (2010).
8. Wasternack, C. & Hause, B. Jasmonates: biosynthesis, perception, signal transduction and action in plant stress response, growth and development. An update to the 2007 review in Annals of Botany. *Ann Bot* **111**, 1021-58 (2013).
9. Schneider, K. *et al.* A new type of peroxisomal acyl-coenzyme A synthetase from Arabidopsis thaliana has the catalytic capacity to activate biosynthetic precursors of jasmonic acid. *J Biol Chem* **280**, 13962-72 (2005).
10. Cruz Castillo, M., Martinez, C., Buchala, A., Metraux, J.P. & Leon, J. Gene-specific involvement of beta-oxidation in wound-activated responses in Arabidopsis. *Plant Physiol* **135**, 85-94 (2004).
11. Ziegler, J. *et al.* Molecular cloning of allene oxide cyclase. The enzyme establishing the stereochemistry of octadecanoids and jasmonates. *J Biol Chem* **275**, 19132-8 (2000).
12. Chini, A. *et al.* The JAZ family of repressors is the missing link in jasmonate signalling. *Nature* **448**, 666-71 (2007).
13. Yang, W. *et al.* AtPLAI is an acyl hydrolase involved in basal jasmonic acid production and Arabidopsis resistance to Botrytis cinerea. *J Biol Chem* **282**, 18116-28 (2007).
14. Lavelle, D.O. Genetics of candidate genes for developmental and domestication-related traits in lettuce. *Dissertations & Theses - Gradworks* **14**, 167-83 (2009).
15. Johnson, W.C. *et al.* Lettuce, a shallow-rooted crop, and Lactuca serriola, its wild progenitor, differ at QTL determining root architecture and deep soil water exploitation. *Theoretical and Applied Genetics* **101**, 1066-1073 (2000).
16. Nishioka, M. *et al.* Mapping of QTLs for Bolting Time in Brassica rapa (syn. campestris) under Different Environmental Conditions. *Breeding Science* **55**, 127-133 (2005).
17. Abbott & Aaron. The isolation of flowering time genes from lettuce to enable the manipulation of bolting time. *Acta Physica Slovaca* **61**, 811-946 (2010).
18. Michaels, S.D. *et al.* AGL24 acts as a promoter of flowering in Arabidopsis and is positively regulated by vernalization. *Plant J* **33**, 867-74 (2003).
19. Fowler, S. *et al.* GIGANTEA: a circadian clock-controlled gene that regulates photoperiodic flowering in Arabidopsis and encodes a protein with several possible membrane-spanning

domains. *EMBO J* **18**, 4679-88 (1999).
20. Lu, S.X., Knowles, S.M., Andronis, C., Ong, M.S. & Tobin, E.M. CIRCADIAN CLOCK ASSOCIATED1 and LATE ELONGATED HYPOCOTYL function synergistically in the circadian clock of Arabidopsis. *Plant Physiol* **150**, 834-43 (2009).
21. Nakamichi, N., Kita, M., Ito, S., Yamashino, T. & Mizuno, T. PSEUDO-RESPONSE REGULATORS, PRR9, PRR7 and PRR5, together play essential roles close to the circadian clock of Arabidopsis thaliana. *Plant Cell Physiol* **46**, 686-98 (2005).
22. Farinas, B. & Mas, P. Functional implication of the MYB transcription factor RVE8/LCL5 in the circadian control of histone acetylation. *Plant J* **66**, 318-29 (2011).
23. Garcia, D., Fitz Gerald, J.N. & Berger, F. Maternal control of integument cell elongation and zygotic control of endosperm growth are coordinated to determine seed size in Arabidopsis. *Plant Cell* **17**, 52-60 (2005).
24. Wang, A. *et al.* The VQ motif protein IKU1 regulates endosperm growth and seed size in Arabidopsis. *Plant J* **63**, 670-9 (2010).
25. Eng, R.C. & Wasteneys, G.O. The microtubule plus-end tracking protein ARMADILLO-REPEAT KINESIN1 promotes microtubule catastrophe in Arabidopsis. *Plant Cell* **26**, 3372-86 (2014).
26. Chen, Q. *et al.* The basic helix-loop-helix transcription factor MYC2 directly represses PLETHORA expression during jasmonate-mediated modulation of the root stem cell niche in Arabidopsis. *Plant Cell* **23**, 3335-52 (2011).
27. Li, M., Qin, C., Welti, R. & Wang, X. Double knockouts of phospholipases Dzeta1 and Dzeta2 in Arabidopsis affect root elongation during phosphate-limited growth but do not affect root hair patterning. *Plant Physiol* **140**, 761-70 (2006).
28. Belitz, H.D., Grosch, W., Schieberle, P. & SpringerLink (Online service). Food Chemistry. 4th rev. and extended edn (Springer-Verlag,, Berlin, 2009).
29. Ajjawi, I., Rodriguez Milla, M.A., Cushman, J. & Shintani, D.K. Thiamin pyrophosphokinase is required for thiamin cofactor activation in Arabidopsis. *Plant Mol Biol* **65**, 151-62 (2007).
30. Moccand, C. *et al.* The pseudoenzyme PDX1.2 boosts vitamin B6 biosynthesis under heat and oxidative stress in Arabidopsis. *J Biol Chem* **289**, 8203-16 (2014).
31. Laing, W.A., Wright, M.A., Cooney, J. & Bulley, S.M. The missing step of the L-galactose pathway of ascorbate biosynthesis in plants, an L-galactose guanyltransferase, increases leaf ascorbate content. *Proc Natl Acad Sci U S A* **104**, 9534-9 (2007).
32. Kanwischer, M., Porfirova, S., Bergmuller, E. & Dormann, P. Alterations in tocopherol cyclase activity in transgenic and mutant plants of Arabidopsis affect tocopherol content, tocopherol composition, and oxidative stress. *Plant Physiol* **137**, 713-23 (2005).
33. Gobert, A., Isayenkov, S., Voelker, C., Czempinski, K. & Maathuis, F.J. The two-pore channel TPK1 gene encodes the vacuolar K+ conductance and plays a role in K+ homeostasis. *Proc Natl Acad Sci U S A* **104**, 10726-31 (2007).
34. Zhu, J.K. Regulation of ion homeostasis under salt stress. *Curr Opin Plant Biol* **6**, 441-5 (2003).
35. Gaymard, F. *et al.* Identification and disruption of a plant shaker-like outward channel involved in K+ release into the xylem sap. *Cell* **94**, 647-55 (1998).
36. Dkhar, J. & Pareek, A. What determines a leaf's shape? *Evodevo* **5**, 47 (2014).
37. Cheng, F. *et al.* Subgenome parallel selection is associated with morphotype diversification and convergent crop domestication in Brassica rapa and Brassica oleracea. *Nat Genet* (2016).
38. Liang, J., Liu, B., Wu, J., Cheng, F. & Wang, X. Genetic Variation and Divergence of Genes

Involved in Leaf Adaxial-Abaxial Polarity Establishment in Brassica rapa. *Front Plant Sci* **7**, 94 (2016).

39. Tsukaya, H. Leaf development. *Arabidopsis Book* **11**, e0163 (2013).
40. Kalve, S., De Vos, D. & Beemster, G.T. Leaf development: a cellular perspective. *Front Plant Sci* **5**, 362 (2014).
41. Gomez-Mena, C. & Sablowski, R. ARABIDOPSIS THALIANA HOMEOBOX GENE1 establishes the basal boundaries of shoot organs and controls stem growth. *Plant Cell* **20**, 2059-72 (2008).
42. Miyashima, S., Sebastian, J., Lee, J.Y. & Helariutta, Y. Stem cell function during plant vascular development. *EMBO J* **32**, 178-93 (2013).
43. Zhong, R. & Ye, Z.H. Secondary cell walls: biosynthesis, patterned deposition and transcriptional regulation. *Plant Cell Physiol* **56**, 195-214 (2015).
44. Li, S., Bashline, L., Lei, L. & Gu, Y. Cellulose synthesis and its regulation. *Arabidopsis Book* **12**, e0169 (2014).
