# Supplementary material for: RNA sequencing provides insights into the evolution of lettuce and the regulation of flavonoid biosynthesis
Source: Nat Commun. 2017 Dec 22;8:2264. doi: 10.1038/s41467-017-02445-9 (PMC5741661; doi:10.1038/s41467-017-02445-9)
Supplement: Supplementary file 3 — Description of Additional Supplementary Files [file 41467_2017_2445_MOESM3_ESM.pdf]

Description of Supplementary Files

File Name: Supplementary Data 1
Description: Summary of the 240 *Lactuca* accessions

File Name: Supplementary Data 2
Description: List of SNPs that have a high impact on gene function across groups/types

File Name: Supplementary Data 3
Description: Selective sweeps detected between *L. serriola* and cultivated lettuce

File Name: Supplementary Data 4
Description: Detection of positive selection in butterhead

File Name: Supplementary Data 5
Description: Detection of positive selection in crisphead

File Name: Supplementary Data 6
Description: Detection of positive selection in romaine

File Name: Supplementary Data 7
Description: Detection of positive selection in stem

File Name: Supplementary Data 8
Description: Introgressed regions from *L. serriola* in cultivated lettuce

File Name: Supplementary Data 9
Description: Introgressed regions from *L. saligna* in cultivated lettuce

File Name: Supplementary Data 10
Description: Introgressed regions from *L. virosa* in cultivated lettuce

File Name: Supplementary Data 11
Description: List of identified eQTLs

File Name: Supplementary Data 12
Description: List of identified distant eQTL hotspots

File Name: Supplementary Data 13
Description: List of candidate master regulators and their targets

File Name: Supplementary Data 14
Description: Genes associated with flavonoid biosynthesis in lettuce

File Name: Supplementary Data 15
Description: Other genes possibly involved in flavonoid biosynthesis
